# Supplementary material for: Honest signaling in academic publishing
Source: PLoS One. 2021 Feb 23;16(2):e0246675. doi: 10.1371/journal.pone.0246675 (PMC7901761; doi:10.1371/journal.pone.0246675)
Supplement: S1 File — (DOCX) [file pone.0246675.s002.docx]

**Honest signaling in academic publishing**

Leonid Tiokhin*^1^, Karthik Panchanathan^2^, Daniel Lakens^1^, Simine Vazire^3^, Thomas Morgan^4^, Kevin Zollman^5^

^1^Human Technology Interaction Group, Eindhoven University of Technology, P.O. Box 513, 5600 MB, Eindhoven, the Netherlands

^2^Department of Anthropology, University of Missouri, USA

^3^Department of Psychology, University of California, Davis, USA

^4^School of Human Evolution and Social Change, Arizona State University, USA

^5^Department of Philosophy, Carnegie Mellon University, USA

*Corresponding author

Email: leotiokhin@gmail.com

**Modifying the probability that low-ranking journals accept submitted papers**

Our models assume that low-ranking journals accept all submitted papers. Below, we modify this assumption, using the simple model in the main text as an illustration. As in the main text, assume that high-ranking journals accept and reject high-quality papers with probabilities *P_H_* and (1 – *P_H_*), and accept and reject low-quality papers with probabilities *P_L_* and (*1 – P_L_*). Also assume that low-ranking journals accept and reject high-quality papers with probabilities *P_h_* and (1 – *P_h_*), and accept and reject low-quality papers with probabilities *P_l_* and (*1 – P_l_*).

A high-quality paper is worth submitting to a high-ranking journal instead of a low-ranking journal when:

$$BP_{H}+\left( 1-P_{H} \right)(bP_{h})>bP_{h}$$

$$\begin{aligned} P_{H}\left( B-{bP}_{h} \right)>0\#\left( S1 \right) \end{aligned}$$

A low-quality paper is worth submitting to a high-ranking journal instead of a low-ranking journal when:

$$BP_{L}+\left( 1-P_{L} \right)(bP_{l})>bP_{l}$$

$$\begin{aligned} P_{L}\left( B-{bP}_{l} \right)>0\#\left( S2 \right) \end{aligned}$$

Conditions S1 and S2 above are satisfied if a scientist can publish papers in high-ranking journals with non-zero probabilities, and if high-ranking publications are worth more than low-ranking ones. When low-ranking journals sometimes reject submissions, this reduces the expected value of low-ranking submissions. This is equivalent to reducing the value of *b*. As such, both conditions can also be satisfied if high- and low-ranking publications are equally beneficial, as long as low-ranking submissions are rejected with non-zero probabilities.

**Costs for low-ranking submissions**

Our model analyzes a case were low-ranking submissions are cost free (*c = 0*). Below, we modify this assumption, using the simple model in the main text as an illustration. As in the main text (Eq. 5) assume that scientists pay a cost, *C*, to submit a paper to a high-ranking journal. Also assume that scientists pay a cost *c*, to submit a paper to a low-ranking journal, where *C > c > 0*. As in the main text, assume that high-ranking journals accept and reject high-quality papers with probabilities *P_h_* and (1 – *P_h_*), and accept and reject low-quality papers with probabilities *P_l_* and (*1 – P_l_*). Assume that low-ranking journals accept all submitted papers.

A high-quality paper is worth submitting to a high-ranking journal instead of a low-ranking journal when:

$$BP_{h}-C+\left( 1-P_{h} \right)\left( b-c \right)>b-c$$

$$\begin{aligned} P_{h}\left( B-b+c \right)>C\#\left( S3 \right) \end{aligned}$$

A low-quality paper is worth submitting to a high-ranking journal instead of a low-ranking journal when:

$$BP_{l}-C+\left( 1-P_{l} \right)\left( b-c \right)>b-c$$

$$\begin{aligned} P_{l}\left( B-b+c \right)>C\#\left( S4 \right) \end{aligned}$$

Following the derivation for Eq. 5 in the main text, a separating equilibrium, such that scientists only submit high-quality papers to high-ranking journals, exists when:

$$\begin{aligned} P_{h}>\frac{C}{B-b+c}>P_{l}\#\left( S5 \right) \end{aligned}$$

Because low-ranking journals accept all papers, the payoff for low-ranking submissions is always *b - c*. As such, provided that *b > c > 0*, adding low-ranking submission costs is equivalent to reducing the value of *b* (*b* can alternatively be interpreted as the net payoff to publishing in low-ranking journals, taking cost into account). This means that the specific values of *b* and *c* are immaterial.

**Differential low-ranking acceptance probabilities and costly submission to low-ranking journals**

As above, assume that scientists pay a cost, *C*, to submit a paper to a high-ranking journal and pay a cost, *c*, to submit a paper to a low-ranking journal, where *C > c > 0*. Assume that high-ranking journals accept and reject high-quality papers with probabilities *P_H_* and (1 – *P_H_*), and accept and reject low-quality papers with probabilities *P_L_* and (*1 – P_L_*). Assume that low-ranking journals accept and reject high-quality papers with probabilities *P_h_* and (1 – *P_h_*), and accept and reject low-quality papers with probabilities *P_l_* and (*1 – P_l_*).

A high-quality paper is worth submitting to a high-ranking journal instead of a low-ranking journal when:

$$BP_{H}-C+\left( 1-P_{H} \right)(bP_{h}-c)>bP_{h}-c$$

$$\begin{aligned} P_{H}\left( B-bP_{h}+c \right)>C\#\left( S6 \right) \end{aligned}$$

A low-quality paper is worth submitting to a high-ranking journal instead of a low-ranking journal when:

$$BP_{L}-C+\left( 1-P_{L} \right)(bP_{l}-c)>bP_{l}-c$$

$$\begin{aligned} P_{L}\left( B-bP_{l}+c \right)>C\#\left( S7 \right) \end{aligned}$$

A separating equilibrium, such that scientists only submit high-quality papers to high-ranking journals, exists when:

$$\begin{aligned} P_{H}\left( B-bP_{h}+c \right)>C>P_{L}\left( B-bP_{l}+c \right)\#\left( S8 \right) \end{aligned}$$

If low-ranking journals accept all papers (*P_h_ = P_l_* *= 1*), the condition for the separating equilibrium is identical to Eq. S5. As low-ranking journals become relatively more likely to accept low- versus high-quality papers (*P_l_ > P_h_*), this increases the range of conditions in which honest submission can exist. If low-ranking journals become relatively more likely to accept high- versus low-quality papers (*P_h_ > P_l_* ), the range of conditions in which honest submission can exist decreases.

**Differential benefits post-publication**

Differential benefits for low- versus high-quality papers published in high-ranking journals can ensure honest submission. This can be seen in the more complex model presented later in the SI. Below, we also illustrate this by modifying the simple model presented in the main text. Assume that scientists receive benefits *B_h_* and *B_l_* for publishing high- and low-quality papers in high-ranking journals, respectively. Assume that high-quality, high-ranking publications are worth more than low-quality, low-ranking ones (*B_h_* *>* *B_l_*). *B_l_* can also be interpreted as the benefit of a low-quality, high-ranking publication, minus post-publication costs (e.g., fewer citations because of failures to replicate).

Following a similar derivation as for Eqs. 3-5 in the main text, a separating equilibrium, such that scientists submit only high-quality papers to high-ranking journals, exists when:

$$\begin{aligned} P_{h}\left( B_{h}-b \right)>C>P_{l}\left( B_{l}-b \right)\#\left( S9 \right) \end{aligned}$$

The conditions for honesty are identical to those of Eq. 5 in the main text, with one exception: honest submission is more likely when high-quality, high-ranking publications are relatively more valuable than low-quality, high-ranking ones (*B_h_* *>* *B_l_*). Honest submission can exist even if journals cannot differentiate between high-and low-quality papers (*P_h_ = P_l_*). This occurs because, regardless of the acceptance probabilities, the cost of high-ranking submission can be smaller the benefit of high-quality, high-ranking publications (*B_h_*), but outweigh the benefit of low-quality, high-ranking ones (*B_l_*).

**A more complex model with both scientists and journals as strategic actors**

We have thus far assumed that scientists strategize in a world where journal behavior is held constant. Below, we extend our model to also allow for strategic behavior on the part of journals. The qualitative insights generated by this model are similar to that of the simpler models presented earlier.

Consider a modified version of the differential benefits (Eq. 5), differential costs (Eq. 8), and resubmission costs (Eq. 11) models in the main text. Assume that a high-ranking publication results in payoffs *B_h_* and *B_l_* for high- and low-quality papers, respectively. Papers directly submitted to and published in the low-ranking journal result in payoffs *b_h_* and *b_l_* for high- and low-quality papers, respectively. To capture the cost of resubmission, we assume that *b_h_* and *b_l_* represent the marginal benefits of direct submission to a low-ranking journal for high- and low-quality papers, respectively, as opposed to submitting first to the high-ranking journal, getting rejected, and only then re-submitting to the low-ranking journal. In other words, papers rejected by the high-ranking journal and subsequently resubmitted to and accepted by the low-ranking journal receive a smaller payoff than papers directly submitted to the low-ranking journal.

Next, upon submission to a high-ranking journal, the scientist may pay an optional submission cost (*C_h_* and *C_l_* for high- and low-quality papers, respectively). This submission cost could be an explicit fee, but could also include other costs imposed on the authors, such as requiring that the paper be formatted in a particular way, or that authors fill out lengthy questionnaires or write bespoke cover letters. While journals can perfectly assess whether the scientist has paid the submission cost, they can only imperfectly determine the quality of the submitted paper. Upon receiving the submission, journals decide whether to send the manuscript to harsh or soft peer-review. “Harsh” peer review can be anything that makes it more difficult to publish (e.g., automatic desk rejection, an increased threshold for acceptance, or choosing reviewers who are known to have higher standards). We assume that, because of the currently widespread norm that papers must be peer-reviewed for a scientific publication to be considered legitimate, journals do not have the option to accept all submitted papers (i.e., the current norms of science act as an exogenous constraint that prevents journals from accepting all papers, even if all submissions are high quality. Note that, in the real world, journals that accept all submissions are considered “predatory”).

Journals have imperfect information about the quality of submitted papers and probabilistically determine submission quality. Assume that, after choosing the harshness of peer review, other aspects of the peer-review process are exogenous to the journal. Peer reviewers are not affected by the choice of the scientist to pay the submission cost, but have some information about the quality of the paper. If a high-ranking journal sends a paper to harsh peer-review, the paper gets accepted with probabilities $\bar{P}$*_h_* and $\bar{P}$*_l_* for high- and low-quality papers, respectively, where $\bar{P}$*_h_* > $\bar{P}$*_l_*. If a high-ranking journal sends a paper to soft peer-review, the paper gets accepted with probabilities $P$*_h_* and $P$*_l_* for high- and low-quality papers, respectively, where $P$*_h_* > $P$*_l_*. Assume that journals do not pay differential costs for subjecting papers to harsh versus soft peer-review.

A high-ranking journal receives a payoff of 1 for publishing a high-quality paper and pays a cost δ for publishing a low-quality paper, where δ ≥ 0. When δ is small, publishing a low-quality paper has a small cost relative to the benefit of publishing a high-quality paper. When δ is large, publishing a low-quality paper is very costly for journals.

S1 Fig depicts the full model.

**S1 Fig. Academic publishing with two-sided strategic interactions.** A decision tree with possible moves by both scientist and journals. In the first move, papers are randomly determined to be high- or low-quality. In the second move, the scientist chooses whether to submit the paper to either the high-ranking journal, with or without paying the submission cost, or to the low-ranking journal. The high-ranking submission cost is *C_h_* and *C_l_* for high-quality and low-quality papers, respectively. In the third move, a high-ranking journal decides whether to send the paper to harsh or soft peer review. Journals have imperfect information about paper quality. When high-ranking journals send papers to harsh peer-review, they accept high- and low-quality papers with probabilities $\bar{P}$*_h_* and $\bar{P}$*_l_*, respectively. When high-ranking journals send papers to soft peer-review, they accept high- and low-quality papers with probabilities $P$*_h_* and $P$*_l_*, respectively. Low-ranking journals accept all submissions. Papers rejected from high-ranking journals are re-submitted to low-ranking journals (not depicted). Dotted lines depict the journal’s information sets. For each node in an information set, the journal does not know at which node they are.

To determine the conditions for a separating equilibrium, we use standard Nash-equilibrium analysis (1) to determine the conditions in which honest submission is the best response of scientists when interacting with journals. To do so, assume that scientists strategize in a world where the common journal strategy is one that only sends papers to soft peer-review when the submission cost has been paid.

If a scientist submits a high-quality paper to a high-ranking journal, the scientist should pay the submission cost when:

$$P_{h}B_{h}-C_{h}>{\bar{P}_{h}B}_{h}$$

$$\begin{aligned} B_{h}\left( P_{h}-\bar{P}_{h} \right)>C_{h}\#(S10) \end{aligned}$$

Similarly, if a scientist submits a low-quality paper to a high-ranking journal, the scientist should pay the submission cost when:

$$P_{l}B_{l}-C_{l}>\bar{P}_{l}B_{l}$$

$$\begin{aligned} B_{l}\left( P_{l}-\bar{P}_{l} \right)>C_{l} \end{aligned}$$

Rewriting this inequality to express low- and high-ranking submission costs in the same units, substitute *C_l_* *=* *kC_h_* , where *k > 1*. The inequality thus becomes:

$$\begin{aligned} B_{l}\left( P_{l}-\bar{P}_{l} \right)>{kC}_{h}\#(S11) \end{aligned}$$

Thus, if a scientist submits to a high-ranking journal, a separating equilibrium exists, such that the scientist only pays the submission cost if submitting high-quality papers when:

$$\begin{aligned} B_{h}\left( P_{h}-\bar{P}_{h} \right)>C_{h}> \frac{B_{l}\left( P_{l}-\bar{P}_{l} \right)}{k}\#\left( S12 \right) \end{aligned}$$

Assuming Eq. S12 is satisfied, a scientist should honestly submit a low-quality paper to a low-ranking journal if the payoff of a direct low-ranking submission is larger than the expected payoff of submitting to a high-ranking journal without paying the submission cost. This occurs when:

$$\begin{aligned} b_{l}>\bar{P}_{l}B_{l}\#(S13) \end{aligned}$$

Further, a scientist should honestly submit a high-quality paper to a high-ranking journal if the expected payoff of a costly submission to a high-ranking journal (and having one’s paper sent to soft peer-review) is larger than the payoff of a direct low-ranking submission. This occurs when:

$$\begin{aligned} P_{h}B_{h}-C_{h}>b_{h}\#(S14) \end{aligned}$$

If Eqs. S12 — S14 are satisfied and journals adopt the specified strategy, a separating equilibrium exists wherein scientists submit high-quality papers to high-ranking journals (and pay the submission cost) and submit low-quality papers to low-ranking journals (see supplementary for analysis of an alternative equilibrium in which authors submit all papers to high-ranking journals but only pay the submission cost for high-quality papers). Note the similarity between Eq. S12 and Eq. 8 in the main text. The range of conditions in which scientists pay the high-ranking submission cost only for high-quality papers increases when 1) high-quality publications in high-ranking journals are worth more than low-quality ones (i.e., differential benefits via large values of *B_h_ – B_l_*), 2) the marginal increase in acceptance probability from harsh to soft peer-review is larger for high-quality papers (i.e., differential benefits via large values of ($P$*_h_ –* $\bar{P}$*_h_*) *–* ($P$*_l_ –* $\bar{P}$*_l_*)), and 3) submitting low- versus high-quality papers to high-ranking journals becomes relatively costlier (i.e., differential costs via large values of *k*).

Next, we establish the conditions in which journals should send submissions to soft peer-review only when scientists have paid the submission cost. Assume that journals strategize in a world where the common strategy among scientists is one of costly honest submission (as established above).

A high-ranking journal prefers to publish high-quality papers and reject low-quality ones. As such, the journal should soft peer-review a paper that has paid the submission cost (i.e., a high-quality paper) when:

$$\begin{aligned} P_{h}>\bar{P}_{h}\#(S15) \end{aligned}$$

Here, no scientist ever submits a high-quality paper to a high-ranking journal without paying the submission cost. As such, the high-ranking journal is indifferent between all possible strategies of responding to scientists who make submissions without paying the submission cost and can freely choose any strategy (e.g., send papers that fail to pay the cost to harsh peer review). Condition S15 is always satisfied. Thus, at equilibrium, journals simply minimize their probability of rejecting high-quality submissions. Note that the journal strategy “soft peer-review only if scientists pay the submission cost” is not a strict Nash equilibrium (see supplementary materials for discussion).

**Notes regarding the more complex model with both scientists and journals as strategic actors**

It is worth noting that the journal strategy “soft peer-review only if scientists pay the submission cost” is not a strict Nash equilibrium. This is because other journal strategies (e.g., “always soft-peer review”) have the same expected payoff at an equilibrium where scientists always engage in costly honest paper submission. We do not find this concerning because, in reality, there will be occasional deviations from equilibrium behavior. For example, if scientists deviate from honestly submitting low-quality papers to low-ranking journals to submitting these papers to high-ranking journals without paying the submission cost, there will be incentives for journals to maintain their equilibrium strategy. But this alone is not sufficient to ensure stability. For example, if some scientists deviate from costly honest submission to submitting high-quality papers to high-ranking journals without paying the submission cost, journals will have incentives to move away from the equilibrium strategy of “soft peer-review only if scientists pay the submission cost”. More formally, we note that while the equilibrium we identify is not strict, it is plausibly a trembling-hand-perfect equilibrium (2).

We also note that, in the more complex model, two types of costs ensure honest paper submission. Initial submission costs ensure that if a scientist submits to a high-ranking journal, the scientist only pays the submission cost if submitting a high-quality paper (Eq. S12). Resubmission costs (i.e., the marginal loss in payoff from being rejected and submitting to the low-ranking journal versus a direct low-ranking submission) ensure that a low-quality paper is not worth submitting to the high-ranking journal (Eq. S13) but a high-quality paper is (Eq. S14). Such a resubmission cost can be conceptualized as anything that reduces the payoff of a paper rejected from a high-ranking journal (e.g., time spent on reformatting; a reduced payoff from publication because other scientists lower their evaluation of your paper based on its past history of rejection or negative reviews).

**Limiting the number of submissions in the more complex model**

As in the main text, it is straightforward to show that limiting the number of submissions can also ensure honest journal submission, regardless of journal strategy, even when submissions are cost free. For illustrative purposes, again consider a modified version of the strategic model where all high-ranking and low-ranking publications are equally beneficial (*B_h_ = B_l_ = B; b_h_ = b_l_ = b),* and there are no submission costs (*C_h_ = C_l_ = 0*). Also assume that scientists can only submit each paper to one journal.

If high-ranking journals send all papers to harsh review, a high-quality paper is worth submitting to a high-ranking journal when:

$$\begin{aligned} b<\bar{P}_{h}B \end{aligned}$$

If high-ranking journals send all papers to harsh review, a low-quality paper is worth submitting to a high-ranking journal when:

$$\begin{aligned} b<\bar{P}_{l}B \end{aligned}$$

A separating equilibrium, such that a scientist submits only high-quality papers to high-ranking journals exists when:

$$\begin{aligned} \bar{P}_{l}B<b<\bar{P}_{h}B\#(S16) \end{aligned}$$

If high-ranking journals send all papers to soft review, the condition for a separating equilibrium will be:

$$\begin{aligned} P_{l}B<b<P_{h}B\#(S17) \end{aligned}$$

If high-ranking journals send high-quality papers to soft review and low-quality papers to harsh review, the condition for a separating equilibrium will be:

$$\begin{aligned} \bar{P}_{l}B<b<P_{h}B\#(S18) \end{aligned}$$

Regardless, the logic by which limiting number of submissions can ensure honesty is the same as that presented in the main text. When the number of submissions is limited, scientists face an opportunity cost because submitting to one journal precludes submission to another. This disincentivizes deceptive submission as long as the expected value of a higher-probability, low-ranking publication outweighs the expected value of a lower-probability, high-ranking one.

**An alternative equilibrium in the more complex model**

In the more complex model in main text, where both authors and journals are strategic actors, we focused on a separating equilibrium in which authors submit high-quality papers to high-ranking journals (and pay the submission cost) and submit low-quality papers to low-ranking journals. However, in a different parameter range, there exists another equilibrium, in which authors submit both high-quality and low-quality papers to high-ranking journals, but only pay the submission cost when submitting the high-quality papers. For this separating equilibrium, it needs to be the case that the condition Eq. S12 is satisfied:

$$\begin{aligned} B_{h}\left( P_{h}-\bar{P}_{h} \right)>C_{h}> \frac{B_{l}\left( P_{l}-\bar{P}_{l} \right)}{k}\# \end{aligned}$$

but that the condition in Eq. 18 in the main text is not satisfied. That is, it needs to be the case that:

$$\begin{aligned} b_{l}<\bar{P}_{l}B_{l}\#(S19) \end{aligned}$$

This would mean that authors receive a higher expected payoff from submitting a low-quality paper to a high-ranking journal and having that paper undergo harsh peer review, than from submitting the low-quality paper directly to a low-ranking journal. Eq. S19 is more likely to be satisfied when 1) low-ranking, low-quality publications are not worth very much (i.e., small values of *b_l_*), 2) low-quality papers that undergo harsh peer review have a high probability of acceptance (i.e., large values of $\bar{P}$*_l_*) and 3) low-quality publications in high-ranking journals receive high payoffs (i.e., large values of *B_l_*).

In this alternative equilibrium when Eq. S19 is satisfied, authors submit all papers to high-ranking journals, but only pay the submission cost when submitting high-quality papers. This allows high-ranking journals to perfectly separate papers based on whether authors paid the submission cost. Because high-ranking journals prefer to publish high-quality papers and reject low-quality ones, high-ranking journals simply send all papers that paid the submission cost to soft peer review, and all papers that did not pay the submission cost to harsh peer review.

**Broader Relevance**

Our paper focused on information asymmetries and conflicts of interest in academic publishing. However, many other domains of academic science share these characteristics. In grant applications, scientists know more about the quality and feasibility of proposed projects than do funders, and can benefit from exaggerating proposal quality. In academic talks, presenters know more about the quality of their research than do audience members, and can benefit from exaggerating the quality of their work to impress audiences. In writing letters of recommendation, recommenders know more about the subject of a letter (e.g., a prospective graduate student or job applicant) than does the letter recipient (e.g., a member of a hiring committee), and recommenders can increase the probability that an applicant is viewed favorably by writing overly-positive letters. Our model provides a tool for thinking about how to ensure honesty in these domains. In the case of grants, if low-quality projects have a sufficiently-lower probability of being funded than high-quality ones, only scientists with high-quality projects will be incentivized to pay the cost of writing proposals. This highlights the importance of improving the reliability of grant-review (3–6). In the case of talks, we can ensure honesty by increasing the probability that scientists who oversell their work are caught (increasing *P_h_ – P_l_*). This can be accomplished in several ways. Presenters could be mandated to adhere to transparent research practices, thereby making the quality of their work more readily apparent. Alternatively, audiences could be seeded with experts in the presenter’s field, as experts possess background knowledge that improves their ability to evaluate the presented research and detect obfuscation. Such experts could also be responsible for asking the tough questions that other audience members may shy away from. And in the case of letters of recommendation, we can mandate detailed statements about candidates’ qualifications, including tangible evidence and a ranking of candidates relative to others. Such statements may be more difficult to write for low- versus high-quality candidates (increasing *C_l_ – C_h_*) and allow letter recipients to better differentiate between candidates of varying quality (increasing *P_h_ – P_l_*).

**References**

1. Gintis H. Game theory evolving: A problem-centered introduction to modeling strategic behavior. Princeton university press; 2000.

2. Selten R. Reexamination of the perfectness concept for equilibrium points in extensive games. International Journal of Game Theory. 1975;4(1):25–55.

3. Fang FC, Bowen A, Casadevall A. NIH peer review percentile scores are poorly predictive of grant productivity. Elife. 2016;5:e13323.

4. Forscher PS, Brauer M, Cox WT, Devine PG. How many reviewers are required to obtain reliable evaluations of NIH R01 grant proposals? 2019;

5. Pier EL, Brauer M, Filut A, Kaatz A, Raclaw J, Nathan MJ, et al. Low agreement among reviewers evaluating the same NIH grant applications. Proceedings of the National Academy of Sciences. 2018;115(12):2952–2957.

6. Sattler DN, McKnight PE, Naney L, Mathis R. Grant peer review: improving inter-rater reliability with training. PLoS One. 2015;10(6):e0130450.
