# Supplementary material for: Honest signaling in academic publishing
Source: PLoS One. 2021 Feb 23;16(2):e0246675. doi: 10.1371/journal.pone.0246675 (PMC7901761; doi:10.1371/journal.pone.0246675)
Supplement: Attachment — Submitted filename: Response to reviewers.docx [file pone.0246675.s003.docx]

**EDITORIAL COMMENTS**

Dear Dr. Tiokhin,

Thank you for submitting your manuscript to PLOS ONE. After careful consideration, we feel that it has merit but does not fully meet PLOS ONE’s publication criteria as it currently stands. Therefore, we invite you to submit a revised version of the manuscript that addresses the points raised during the review process.

As you can see, the paper received two divergent reviews, though both were fundamentally positive about the paper. I too am fundamentally positive about the paper, but some of R1's concerns about the assumptions of the model did occur to me too. However, I am not suggesting that you change your assumptions, merely that you acknowledge their limitations. Of course, you may be persuaded by R1's challenge: in which case, by all means change your assumptions and modify the model--or better yet, run multiple models with slightly different assumptions. I will probably not submit the next version of this paper for further review, but make the decision myself.

We look forward to receiving your revised manuscript.

Kind regards,

Jonathan Jong, PhD
Academic Editor
PLOS ONE

**Response:** We are happy to receive a positive evaluation of our manuscript. We have addressed all of the reviewers' comments in the revised version and have more explicitly acknowledged the model's limitations. We provide detailed responses below.

Sincerely,

Leonid Tiokhin, Karthik Panchanathan, Daniel Lakens, Simine Vazire, Thomas Morgan & Kevin Zollman

**REVIEWER COMMENTS**

**Reviewer #1**

**Comment**: This paper discusses an issue in academic publishing – the information asymmetry between authors and editors – and considers how the logic of costly signaling theory can be applied to ensure that only high-quality papers are submitted to high quality journals. Their model considers two types of papers and two types of journals: high quality and low quality. It argues that in the absent of costs, authors will always be incentivized to submit all papers to high-quality journals, but that low-quality papers being reviewed by or published in high quality journals is a problem. The authors consider how costs to submit papers can change the decision calculus so that rational authors will only submit high-quality papers to high-quality journals.

I found the paper to be an interesting exercise, but I wasn’t particularly convinced by its arguments. The model is very simple and relies a number of assumptions that either rarely hold or, in the case of submission costs, *always* hold, raising questions about the conclusions one can draw. I found the discussion around the model to be cursory and at times naïve, with some occasionally problematic recommendations. That said, I thought the approach was interesting, and I found no major errors in the analysis. Connecting signaling theory to academia is a good idea, and I found there to be useful food for thought in this paper. It could also provide the foundation for more detailed modeling work on the subject. Given the broad appeal of the topic, it seems like PLOS ONE could provide a good home for the paper, pending some considerations of the comments below.

**Response:** We thank Reviewer 1 for their constructive feedback. We agree that our manuscript provides a good foundation for future modeling, makes a useful analogy between signaling and the publication process, and has broad appeal. As described in detail below, we modified our manuscript in light of Reviewer 1's suggestions.

**Comment:** I first want to talk about the basic assumptions of the model. The model assumes that
1) Scientists should be honest
2) Scientists can accurately assess the quality of their research
3) Journal rank is a mark of quality, not specificity.
4) All research must be submitted as a paper.

The focus is on (1), and on mechanisms to enforce honesty. The others are implied and not explored, but I think there are some real issues there. First, the extent to which researchers accurately assess the value of their work is highly questionable. It may also be adaptive to overestimate the value of one’s work – scholars that undervalue their work may be selected against in a competitive institutional system.

**Response:** We agree with Reviewer 2 in that it is possible that researchers cannot always accurately assess the value of their work. However, we do not agree that this is either true in general or an established fact. The purpose of our paper is to explore the consequences of assuming that authors can, to some degree, accurately assess the quality of their research. In addition, we now discuss Reviewer 1's comment as a potential future extension of the model (lines 560–561).

Second, the distinction between high- and low-quality journals seems very artificial. Certainly some journals are known to be crap (e.g. some predatory journals), and some are very prestigious, but there are also many journals that are excellent in terms of editorial curation and reputation but somewhat narrow in scope – what are often called technical or specialty journals. The dilemma many authors face is whether their paper has broad enough appeal to be submitted to a fancy interdisciplinary journal like Nature or a more solid but niche society journal.

**Response:** This comment reflects a misunderstanding on the part of Reviewer 1. Reviewer 1 notes that we model "high- and low-quality journals". However, we actually model high- and low-*ranking* journals, wherein authors receive a higher payoff for publishing in higher-ranking journals than lower-ranking journals. There is extensive empirical evidence (see bel0w) that journals vary in rank, that authors prefer to publish in high-ranking journals, and that publishing in higher-ranking journals results in larger career-payoffs for authors. As Reviewer 1 points out, there are many other dimensions along which journals differ (e.g., breadth, scope, etc.) that we do not examine here.

We have now incorporated a discussion of this point into our paper (line 477):

*“Just as experiments simplify reality to clearly establish cause-and-effect relationships, models simplify reality to serve as useful “thinking aids” for scientists (35–37). As with all models, our analysis ignores many real-world details. For example, we do not address other factors that authors may consider when deciding where to submit a paper (e.g., whether a paper is a better fit for an interdisciplinary or specialty journal), although our model is generally relevant to cases where journals vary in selectivity and authors receive different payoffs for publishing in different journals.”*

Van Dijk, D., Manor, O., & Carey, L. B. (2014). Publication metrics and success on the academic job market. *Current Biology*, *24*(11), R516-R517.

McKiernan, E. C., Schimanski, L. A., Nieves, C. M., Matthias, L., Niles, M. T., & Alperin, J. P. (2019). Meta-research: use of the journal impact factor in academic review, promotion, and tenure evaluations. ELife, 8, e47338.

Calcagno, V., Demoinet, E., Gollner, K., Guidi, L., Ruths, D., & de Mazancourt, C. (2012). Flows of research manuscripts among scientific journals reveal hidden submission patterns. Science, 338(6110), 1065-1069.

**Comment**: Third, the authors write that in their ideal scenario, “Scientists would then submit high-quality research to high-ranking journals and low-quality research to lower-ranking ones” (lines 9-10). But why? Why publish low-quality research at all, especially if it’s readily identifiable as low quality? I kept thinking about this throughout the analysis, especially in terms of the cost tradeoffs. It seems like a crucial but missing component was the decision to not submit anything at all. This would carry no cost (except maybe the sunk cost of having done the work) and provide no benefit. This also speaks to something that is glossed over but important. ALL paper submissions incur some cost. It is not trivial to write up the paper and give up time to have the paper under review. The fact that most journals require papers to not be under submission elsewhere is a real tangible cost for all submissions.

**Response:** Reviewer 1 argues that "a crucial but missing component was the decision to not submit anything at all." However, while we understand Reviewer 1's comment, we do not agree with the implication and explain why below. As other readers may see things similarly to Reviewer 1, we have modified the manuscript as described below.

Although the manuscript describes the choice facing scientists as being between high- and low-ranked journals, it could just as equally be presented as a choice between submitting or not submitting work. This is because our model assumes that low-ranked journals accept all papers producing a static payoff (*b*-*c*), which can alternatively be described as the fixed payoff to cutting your losses and not submitting the work at all. We now make this clear (see lines 124 - 127).

*"Further, as authors receive a fixed payoff for low-ranking submissions, this	payoff can also be interpreted as the fixed payoff to an author who cuts their	losses and chooses not to submit their work for publication. "*

Reviewer 1 suggests that our model neglects that fact that "ALL paper submissions incur some cost,” on the basis that we assume *c* = 0. However, this claim is incorrect - in the supplemental materials, we prove that the qualitative results are identical in a model where c > 0 (i.e. all submission do incur a cost, see supplementary pages 1 and 2). The explanation is that "provided that *b > c > 0*, adding low-ranking submission costs is equivalent to reducing the value of *b* (*b* can alternatively be interpreted as the net payoff to publishing in low-ranking journals, taking cost into account). This means that the specific values of *b* and *c* are immaterial."

We have now rephrased the main paper to make this clear (see lines 217-218).

*"The qualitative results are identical in cases where low-ranking submissions	have a non-zero cost (see supplementary materials)."*

**Comment**: Another assumption made is that “high-quality” journals are always incentivized to publish quality research. However, they are also incentivized to publish things that will get attention, and to minimize the risk of needing a retraction due to quality. This probably correlates with quality as the authors describe it, but not always – it would be good to address this, since the model strongly relies on this assumption and might run into problems if journal editors are also acting strategically.

**Response:** It is possible that journals sometimes publish articles just because those papers draw attention and that these papers are not readily evaluated as high quality or low quality (e.g., subjective opinion pieces). Our model does not apply to such papers.

Reviewer 1 asks whether our model "might run into problems if journal editors are also acting strategically." As we note in the manuscript, the qualitative results remain identical in this case (lines 128-134). We prove this in the supplementary materials (pages 4-8) in the section titled " A more complex model with both scientists and journals as strategic actors". The reason that the results are identical is that the key mechanisms by which honesty is ensured (i.e., differential benefits and differential costs) occur by changing incentives for authors, not for journals.

**Comment**: The authors main conclusions are that high quality journals should impose (or continue to impose) costs for submission to dissuade trivial submission of low-quality papers. There are a few concerns about this. First, as noted, all submission is at least somewhat costly. Second, journals have desk rejection, which is a relatively low-cost way to weed out the more low-quality papers. And third, and most importantly, imposing high costs to submission has a potential social consequence, which is that it will tend to preferentially favor researchers who can afford to pay such costs (and so for whom the costs are effectively less). This favors senior researchers at wealthy, prestigious institutions doing normal paradigmatic science. This seems to me to be a major downside and needs to be addressed in the authors’ discussion.

**Response:** Reviewer 1 states that our main conclusions are that "high quality journals should impose (or continue to impose) costs for submission to dissuade trivial submission of low-quality papers." However, our discussion of this point is more nuanced. For instance, we explicitly state that “Our models do not address whether submission costs harm or benefit science.” Instead, we show that submission costs have the *causal effect* of dissuading the submission of low-quality papers to high-impact journals. We also provide an extensive discussion of whether or not this a good idea in practice in the discussion:

"*Our models do not address whether submission costs harm or benefit science.	In biological signaling theory, it is well-established that the high signal costs	necessary for a separating equilibrium can leave all parties worse off than in	a system with lower signal costs and partial or complete pooling of	signals across types [33,66]. Given the difficulty of optimally calibrating	submission costs, future work could extend our analysis to determine what	combination of honesty and submission cost would lead to the most	desirable scientific outcomes (see [53])."*

We have responded to Reviewer 1’s first concern that "all submission is at least somewhat costly", as described above.

Reviewer 1’s second concern is that "journals have desk rejection, which is a relatively low-cost way to weed out the more low-quality papers." We agree, and in the supplementary materials, we show that our qualitative results are identical in a case where journals have the option to make papers more difficult to publish by engaging in behaviors such as automatic desk rejection or choosing reviewers with higher quality standards. We now link to this more explicitly from the main manuscript:

"*The screening process that makes high-quality papers have a higher	acceptance probability could occur via any number of mechanisms (e.g.,	editor screening; peer-reviewer evaluation)."*

Reviewer 1’s third concern is that "imposing high costs to submission has a potential social consequence, which is that it will tend to preferentially favor researchers who can afford to pay such costs" and suggests that we address this in our discussion. We agree that this is an important point, and dedicate a paragraph in the discussion that explicitly addresses this:

“*It is also worth noting that submission costs can exacerbate inequalities, as submissions may be costlier for individuals with fewer resources (e.g., early-career researchers, scientists in developing countries). One solution is to make submission costs conditional on scientists’ ability to pay them (44). This might mean faster review times for early-career researchers or lower submission fees for scientists who have limited funding. “*

_______OTHER COMMENTS_______

**Comment**: The section “Relation to existing models in economics” is useful but probably belongs much earlier in the paper, before the detailed model description. Also, an important but missing reference the authors might consider engaging with is: Crawford and Sobel (1982) Strategic information transmission. Econometrica 50.

**Response:** We have now added a reference to Crawford and Sobel (1982) in the "Relation to existing models in economics" section.

Although we previously had this section in the beginning of the paper, many readers found this section hard to follow without first having the context of our model and its assumptions. However, to improve the flow of the paper, we have moved this section upwards, directly after the model but before the Implications section.

**Comment**: In the description of high vs. low quality research, they write (lines 112-116):“A high-quality paper might be one that thoroughly describes prior research, is methodologically rigorous, conducts appropriate statistical analyses and sensitivity checks, honestly reports all analyses and measures (e.g., no p-hacking or selective reporting of positive results), and clearly distinguishes between exploratory and confirmatory findings (see (5) for other factors that affect research quality). In contrast, a low-quality paper may have fewer or none of these qualities.”

I would argue that you can have low quality research that has all these properties but fails to ask meaningful or important questions. This isn’t just about impact, it’s about insight. Indeed, many papers are likely rejected from “high quality journals” not because the research isn’t rigorous, but because they fail to be asking probing questions in a sufficiently deep fashion. Also, something to think about might be the fact that some research (often but not always “high quality”) may itself be more costly to conduct, and therefore authors may feel “entitled” to publishing in more prestigious journals to justify the investment, while they may feel more comfortable publishing “low-quality” research in third-tier journals because it doesn’t represent a substantial investment.

**Response:** Reviewer 1 notes that research quality is determined by other factors, in addition to those that we focus on in our paper (e.g., research that "fails to ask probing questions in a sufficiently deep fashion").

Our model explicitly focuses on aspects of papers that are under researchers’ control, for which there are information asymmetries between researchers and journals, and for which there are incentives to deceive. We do this both for tractability and because there are larger information asymmetries for aspects of study design than for other parameters such as whether the researcher asked a deep question (e.g., it is easier for the editor to tell whether the author asked a deep question than whether the author ran several studies and measured several dependent variables but only reported a subset of them in the paper). That said, although we use a working definition of quality that considers only aspects of study design, we provide an extensive discussion of the fact that, in reality, journals also prefer papers with a variety of other characteristics (e.g., novelty, deep questions) and discuss the extent to which making alternative assumptions (e.g., that papers vary on more than one dimension) would alter our results:

*"For example, scientists have an incentive to publish each paper in a high-	ranking journal, but high-ranking journals prefer to publish only a subset of	papers (e.g., those with rigorous	methods, compelling evidence, or novel	results)."*

*"Further, we focus on papers varying in only quality, whereas papers and	their perceived value actually vary along many dimensions (e.g., novelty, type	of result (positive or negative), whether conclusions support scientists’	preconceptions [6,68,90]). That said, our models are general enough to	accommodate alternative interpretations of the single “quality” parameter.	For example, we could have described papers as containing either positive or	negative results, journals as preferentially accepting positive results, and	authors as preferring to get all results published in high-ranking journals. If	there were differential benefits to positive versus negative results, there would	be some submission cost at which authors would only benefit from submitting	positive results to high-ranking journals. It is worth noting that some	emerging publishing formats, such as Registered Reports [91], ameliorate this	issue by ensuring results-blind evaluation of submissions. More generally,	future reforms would benefit from considering how publishing norms and	incentives vary for different types of research and across different	scientific	fields. "*

We have now modified the manuscript to refer readers to this discussion section from the model-description section (line 100).

Reviewer 1 also notes that researchers may feel "entitled" to publishing in more prestigious journals to justify their past investment in the paper. However, this would constitute a "sunk cost fallacy" on the part of researchers, because the optimal submission behavior does not depend on a researcher's past investment in the research - it only depends on the expected payoffs of potential actions. As such, it is not within the scope of our model. Addressing this point, we note in the paper that “our models assume that papers vary in quality but do not address the process that generates different types of papers.”

**Comment**: Lines 144-145: “papers are randomly determined to be high- or low-quality.”In reality, there may be many more low quality than high quality papers. Would a skewed ratio change the calculus?

**Response:** No this wouldn't change the model, because we model authors' optimal decisions conditional on the quality of paper that they have. Regardless of whether 1 out of 100 papers or 99 out of 100 papers are high-quality, authors have the same optimal submission strategies in our model. We now indicate this in the main text:

*"Because submission is conditioned on paper type, the proportion of high- or	low-quality papers is not relevant to our model."*

**Comment**: Lines 165: “This illustrates a key conflict of interest in academic publishing.” The conflict here is that without costs, scientists are always incentivized to submit everything to “high-quality” journals, but HQ journals only want to publish HQ work. This being a conflict is contingent on a number of assumptions holding, including the absence of costs. But as noted above, there are almost always costs. There are costs to writing up results, for example, so another option is to just not submit. Consider this paper, which found that most null results are never even written up: Franco et al. (2014) Publication bias in the social sciences: Unlocking the file drawer. Science 345.

**Response:** As we explain above and prove in the supplementary materials, allowing for all submissions to have some cost does not qualitatively change model results because it results in a mathematically equivalent model. We have now rephrased the main paper to make this clear (see response above).

**Comment**: This comment also impinges on the assumption to make the cost of submission to LQ journals c = 0, which they say “does not affect our model’s generality.” (line 179). This is only because the choice is between submit high or submit low. If another option is submit nowhere, which imposes no cost and causes no benefit, there might be cases in which a strategy could be “go big or go home” — submit to high-ranking journal, and if rejected, submit nowhere. This reduces the generalizability of c = 0.

**Response:** As discussed above, meaningfully adding a third option complexifies our model without fundamentally changing its structure and so we do not think it will provide any additional insights. Further, as scientists receive a fixed payoff for low-ranking submissions, this payoff can also be interpreted as the fixed payoff to a scientist who cuts their losses and chooses not to submit their work for publication.

We now discuss this point in our manuscript (see lines 125 - 127).

**Comment**: In terms of deriving values of C to separate HQ and LQ papers, “The key insight is that imposing costs can promote honesty” (line 200). Maybe, but it also seems likely calibrating those costs will often be quite difficult. Especially since the costs and benefits are not constant for all individuals, but vary by research career stage, current prestige of position, workflow to submit and re-submit, etc. Further, if P_h is similar to P_l, there is a very narrow range of C, which interplays with the variation between individuals on both B and C.

**Response:** We have modified the discussion to note that there are challenges with calibrating publishing costs:

*“Given the difficulty of optimally calibrating submission costs, future work could extend our analysis to determine what combination of honesty and submission cost would lead to the most desirable scientific outcomes (see (58)).”*

We also provide an extensive discussion of the interplay between costs, acceptance probabilities, and honest paper submission (lines 197 - 212):

*“Honesty is possible when the cost of high-ranking submission, C, is larger than the expected added benefit of submitting a low-quality paper to a high-ranking journal, P_l_ (B – b), but smaller than the expected added benefit of submitting a high-quality paper a to high-ranking journal, P_h_ (B – b). If As high-ranking publications are become worth much more than low-ranking publications (larger values of B – b), larger submission costs are required to ensure honest submission; otherwise, scientists are will be tempted to submit all papers to high-ranking journals. However, if submission costs are too large, no separation exists, as no paper is worth submitting to a high-ranking journal. As journals become better able to differentiate high- from low-quality papers (larger values of P_h_ – P_l_), the range of conditions under which honesty can exist becomes larger. Consider a case where high-ranking journals accept most high-quality papers and reject most low-quality ones. A scientist who submits low-quality papers to high-ranking journals pays a cost for each submission, but rarely obtains a high-ranking publication. As a result, dishonesty is not worthwhile unless the rare high-ranking publication is extremely valuable. In an extreme case where journals cannot tell the difference between high- and low-quality papers (P_h_ = P_l_), honest submission cannot exist, because scientists lack any incentive to condition journal submission on paper quality. “*

**Comment**: Lines 207-209: “If high-ranking publications are worth much more than low-ranking publications (large values of B – b), large submission costs are required to ensure honest submission; otherwise, scientists are tempted to submit all papers to high-ranking journals.” Not necessarily, if P_h is low. In that case, costs could still be low. Perhaps this suggests that HQ journals should just be more selective?

**Response:** We have now re-worded this section to indicate that this statement is true if all else is equal (i.e., for any value of P_H, as high-ranking publications become worth more, larger submission costs are required to ensure honest submission). We thank Reviewer 1 for pointing out that this point needed clarification.

*“As high-ranking publications become worth much more than low-ranking publications (larger values of B – b), larger submission costs are required to ensure honest submission; otherwise, scientists will be tempted to submit all papers to high-ranking journals. “*

**Comment**: Regarding limiting the number of submissions, they cite suggestions to limit scientists’ lifetime number of publications or to limit scientists to one publication per year (lines 289-291). This example isn’t really appropriate, because it’s talking about limiting the number of publications, not the number of submissions.

**Response:** We cite three papers in this section. The first paper (Azar 2006) specifically proposes limiting scientists' number of submissions. The other two papers make proposals that are about other types of limits. We have now clarified this section to indicate that the Azar paper is specifically about submission limits, whereas the other two paper are merely "related" proposals.

**Comment**: In the Implications, the authors first reiterate the main conclusion: “the costs associated with publishing reduce the incentive to submit low-quality research to high-ranking journals” (lines 334-336). They seem to have ignored desk rejection, which is the main weapon that journals have against wasting time with low-quality submissions. In truth, that might be enough. You haven’t talked at all about decision calculus for the journals. Why should they become more or less selective? What criteria should they use to select papers?

**Response:** As noted in our response to Reviewer 1's previous comment (above), desk rejection does not qualitatively affect our results. We prove this in the supplementary materials, where we extend our model to allow for strategic interaction.

**Comment**: Lines 337-339: “If the benefits of high-ranking publications remained large, scientists would have even larger incentives to submit low-quality research to high-ranking journals, because the costs of doing so would be trivial.” An interesting counterexample might be Sociological Science, which guarantees up/down decisions in 30 days and does not allow major revisions. Their rejection rate is high, and they have established a good reputation.

**Response:** This is a neat example. However, the argument that follows from our model is that a directional reduction in submission costs would cause more low-quality papers to be submitted to high-ranking journals, *all else equal*. The example of Sociological Science is not a good fit here - while they reduced submission costs (reducing turnaround time) they also lowered acceptance probability (no major revisions).

**Comment**: Lines 405-406: “When submissions and resubmissions are cost free…”
Again, I think this is almost never the case in practice. Especially if there’s the additional choice of not submitting at all.

**Response:** We have rewritten this sentence to clarify that, as submission and resubmission costs are reduced, scientists have greater incentives to submit all papers to high-ranking journals regardless of quality (see response above).

**Comment**: Lines 409-411: “If journals preferentially reject low-quality papers, editors can wait some time before sending authors “reject” decisions, thereby causing disproportionate delays for low-quality submissions.” Jesus Christ. No. Editors that do this should be punched in their stupid faces.

**Response:** Perhaps, but editors may feel the same about authors who submit egregiously low-quality work! Punching aside, we have modified this section to indicate that such a reform would, *in principle*, ensure honest submission by introducing resubmission costs, as has previously been argued based on related models in economics.

*"In principle, such costs could be implemented in several ways. If journals	preferentially reject low-quality papers, editors could wait before sending	authors “reject” decisions, thereby causing disproportionate delays for	low-quality submissions [39]."*

**Comment**: Lines 424-426: “Limiting the number of times that papers can be submitted or rejected…” Beyond this being an impossible level of top-down control and overly paternalistic, such a limitation already occurs in practice because most journals have a policy that papers shouldn’t be submitted elsewhere.

**Response:** Reviewer 1's comment here is incorrect – while the policy that "papers shouldn't be submitted elsewhere" creates resubmission delays (i.e., submitting a paper to one journal means a some wait time before the next submission), it does not limit the total number of times that papers can be submitted, because the same paper can be submitted to an infinite number of journals sequentially

We also disagree that limiting the number of submissions is impossible. As we explain in the paper, such a reform simply requires unique identifies for papers (which already exist) and some coordination among journals in a field. For these reasons, this reform has previously been suggested in the economics literature (see Azar, 2006).

**Comment**: Lines 472-473: “we note several extensions.”
Rather than simply listing extensions, which is about what you could have done but didn’t, why not spend some more time talking about the limitations and caveats of your conclusions based on your current model’s assumptions?

**Response:** We now explicitly note the challenges of applying our model to reforming the publishing system in the discussion (line 475) and more explicitly note that "our approach has important limitations" in the introduction (line 97).

**Comment**: As a final note, something I kept asking myself while reading it was: Is this a high quality paper? Is PLOS ONE a high quality journal? Were the authors able to accurately assess the quality of their own research and use their model to help them decide where to submit? This is asked mostly rhetorically, but also seriously to the extent that it forces the question: are there real lessons to be drawn from this?

**Response:**

"Is this a high-quality paper?"

In their review, Reviewer 1 wrote, “*I thought the approach was interesting, and I found no major errors in the analysis. Connecting signaling theory to academia is a good idea, and I found there to be useful food for thought in this paper*.” This was exactly our goal when writing this paper. We are glad our models were accurate to the extent they withstood Reviewer 1’s scrutiny. So, we believe that this is a high-quality paper, under the definition we use in our article.

"Is PLOS ONE a high-quality journal?"

Again, we model journal rank, not journal quality. One of the most common metrics to determine journal rank, both in terms of rewarding researchers and used by researchers to decide where to submit their papers, is the Journal Impact Factor (JIF).

McKiernan, E. C., Schimanski, L. A., Nieves, C. M., Matthias, L., Niles, M. T., & Alperin, J. P. (2019). Meta-research: use of the journal impact factor in academic review, promotion, and tenure evaluations. *ELife*, *8*, e47338.

Calcagno, V., Demoinet, E., Gollner, K., Guidi, L., Ruths, D., & de Mazancourt, C. (2012). Flows of research manuscripts among scientific journals reveal hidden submission patterns. *Science*, *338*(6110), 1065-1069.

Based on JIF, in the multidisciplinary journal category, Plos ONE is ranked 10th out of 141 journals (https://www.scimagojr.com/journalrank.php?area=1000&type=j&category=1000). This places it in a similar ranking with other well-known interdisciplinary journals such as *Royal Society Open Science* and *Scientific Reports*, but below higher-ranking interdisciplinary journals such as *Nature* and *Science*.

"Were the authors able to accurately assess the quality of their own research and use their model to help them decide where to submit?"

Reviewer 1's comment hinges on the assumption that the function of our model is to help make a practical decision, such as where to submit a paper. However, as with any model, our model serves many functions besides just aiding in practical decision making (e.g., Epstein, J. M. (2008). Why model?. *Journal of Artificial Societies and Social Simulation*, *11*(4), 12).

For example, our model 1) shows that existing reforms to lower submission costs will have the unintended consequences of increasing incentives for scientists to submit low-quality work to high-impact journals, 2) makes a novel analogy between signaling theory and academic publishing (and explains how the concepts of differential benefits and differential costs provide conceptual tools for understanding how to change publishing incentives), 3) shows that audits of published papers should target high-impact journals and 4) highlights that limiting the number of submissions can ensure that authors don't submit low-quality papers to high-impact journals (and don't overburden reviewers).

"Are there real lessons to be drawn from this?"

Yes. See 1) above, 2) Reviewer 2, and 3) the Implications section of the paper.

**Reviewer #2**

**Comment:** I really liked this paper. I thought it was clear, simple has a valuable insight, and useful prescriptions.

The basic idea is straightforward (and well explained): there is a fundamental informational and incentives problem in publishing—authors have an incentive to over-sell their work and have information about its value that the readers cannot as easily access, such as how much they had to twist the result or model or citations to make the results sound compelling and novel and interesting. The authors do a good job summarizing some of the inefficiencies this information + incentives problem creates. And then discuss how this problem can be understood and targeted using a standard costly signaling framework. Namely, in order to help readers differentiate between high and low quality papers, its essential that high quality papers can get into higher quality journals with lower relative cost—e.g. by having more attentive or better equipped referees, academic system that gives less credence to bad papers in good journals, or makes journal submission more onerous, misrepresenting results harder, or limits the frequency of submissions. I Think these are all nice insights and valuable prescriptions, and costly signaling proves to be a useful perspective to look at this problem.

Perhaps I missed something significant that other referees will catch, but on my reading, I couldn’t think of anything I would want changed in this paper or any reason to prevent its publication.

**Response:** We are glad that Reviewer 2 found that our manuscript was insightful, clear, and provided valuable insights and useful prescriptions.
